# Modifying the false discovery rate procedure based on the information theory under arbitrary correlation structure and its performance in high-dimensional genomic data

**DOI:** 10.1186/s12859-024-05678-w

**Published:** 2024-02-05

**Authors:** Sedighe Rastaghi, Azadeh Saki, Hamed Tabesh

**Affiliations:** 1 Department of Epidemiology and Biostatistics, School of Health, Mashhad University of Medical Sciences, Mashhad, Iran; 2 Department of Medical Informatics, School of Medicine, Mashhad University of Medical Sciences, Mashhad, Iran

**Keywords:** False discovery rate, Multiple comparison procedures, High-dimensional data, Arbitrary correlation, Conditional fisher information, Efficient Bayesian logistic regression, Entropy

## Abstract

**Background:**

Controlling the False Discovery Rate (FDR) in Multiple Comparison Procedures (MCPs) has widespread applications in many scientific fields. Previous studies show that the correlation structure between test statistics increases the variance and bias of FDR. The objective of this study is to modify the effect of correlation in MCPs based on the information theory. We proposed three modified procedures (M1, M2, and M3) under strong, moderate, and mild assumptions based on the conditional Fisher Information of the consecutive sorted test statistics for controlling the false discovery rate under arbitrary correlation structure. The performance of the proposed procedures was compared with the Benjamini–Hochberg (BH) and Benjamini–Yekutieli (BY) procedures in simulation study and real high-dimensional data of colorectal cancer gene expressions. In the simulation study, we generated 1000 differential multivariate Gaussian features with different levels of the correlation structure and screened the significance features by the FDR controlling procedures, with strong control on the Family Wise Error Rates.

**Results:**

When there was no correlation between 1000 simulated features, the performance of the BH procedure was similar to the three proposed procedures. In low to medium correlation structures the BY procedure is too conservative. The BH procedure is too liberal, and the mean number of screened features was constant at the different levels of the correlation between features. The mean number of screened features by proposed procedures was between BY and BH procedures and reduced when the correlations increased. Where the features are highly correlated the number of screened features by proposed procedures reached the Bonferroni (BF) procedure, as expected. In real data analysis the BY, BH, M1, M2, and M3 procedures were done to screen gene expressions of colorectal cancer. To fit a predictive model based on the screened features the Efficient Bayesian Logistic Regression (EBLR) model was used. The fitted EBLR models based on the screened features by M1 and M2 procedures have minimum entropies and are more efficient than BY and BH procedures.

**Conclusion:**

The modified proposed procedures based on information theory, are much more flexible than BH and BY procedures for the amount of correlation between test statistics. The modified procedures avoided screening the non-informative features and so the number of screened features reduced with the increase in the level of correlation.

**Supplementary Information:**

The online version contains supplementary material available at 10.1186/s12859-024-05678-w.

## Introduction

Controlling the Family Wise Error Rate (FWER) under the nominal level α, in a large-scale multiple testing is an important issue in statistical inference. The simplest method for controlling FWER is a Bonferroni (BF) correction, which can be defined as a modification of the rejection threshold for individual *P*-values. The BF procedure compares all the *p*-values of K simultaneous hypotheses with α/K. This procedure is very conservative and provide a strong control on the FWER and leads to an increase in type II error rate. In most studies, researchers accepted the hazard of the false discoveries to find any possible significance difference [1]. So, the False Discovery Rate (FDR) procedures are proposed and developed. The Benjamini–Hochberg (BH) procedure that compares the *P*-values with a fixed increase in threshold is used in most recent scientific research [2].

The BH procedure is one of the most important methodological advances in testing multiple hypotheses, which has been widely used for screening large data sets of genomic to identify a favorable number of important features. This procedure has an essential assumption of independence between test statistics. However, when dealing with high-dimensional data such as microarray data, genes are usually associated with biological or technical reasons [1, 2].

So, Benjamini-Yekutieli (BY) proposed a simple correction on BH procedure for arbitrary correlation structure. As they reported, this corrected procedure is very conservative [4]. Considering correlation in estimating FDR suggested in several studies [5–17], but to the best of our knowledge, few studies that provide and applicable modification of FDR procedures based on the arbitrary correlation structure.

Initial research in the test of multiple hypotheses and controlling the FDR largely ignored the structure of dependence among the hypotheses, which is often considered a nuisance parameter and is heavily overwhelmed by the assumption of independence [3, 4].

Correlation may lead to more liberal or conservative test methods; therefore, it should be considered in deciding which hypotheses should be reported as alternative hypotheses [5]. Also, the correlation may greatly increase (inflate) the variance of false discoveries and estimators of the common discovery rate [6, 7]. Ignoring the dependence between hypotheses may lead to loss of efficiency and bias in decision-making. On the other hand, errors in non-null distribution can lead to false positive and false negative errors [3]. Consequently, correlation can significantly worsen the performance of many FDR methods [8], and the FDR can be variable if there is a strong correlation [5, 9].

Controlling the FDR under dependency is a major problem that requires a lot of research. The key issue is how to incorporate the dependency structure correctly in the inference. Currently, researchers have focused on the development of multiple comparative methods for the affiliated hypotheses. For the first time, Benjamini and Yekutieli mentioned that the effect of the test statistic dependence on FDR at the level of α is controlled under the desired dependence between *P*-values in the BH procedure. This method is very conservative in practice. They also introduced the concept of positive regression dependence on subsets (PRDS) and proved that the BH procedure controls the FDR for *P*-values with such property [10].

Qiu and Yakovlev showed a strong correlation for FDR only through simulation [7]. Storey et al*.*, Wu, and Clarke and Hall showed that in the asymptotic concept, the BH procedure is valid in poor dependency models, linear process, and Markov dependency [11–13]. Owen and Finner et al*.* showed that the expected values and variance of false-positive cases might have different features under dependence, but results did not provide an FDR, indicating that the BH procedure under severe dependence and variation is vulnerable [6, 14].

Efron and Schwartzman and Lin showed that strong correlations reduce the accuracy of estimating and testing [2, 5]. Specifically, positive or negative correlations have affected the experimental zero distributions of Z-values, which has a significant effect on the subsequent analysis.

The studies carried out by Sun and Tony Cai, and Sun and Wei, and Benjamini and Heller showed that the combination of functional, spatial, and temporal correlations in inference could improve the strength and interpretation of existing methods. However, these methods do not apply to general dependency structures [4, 15, 16]. Also, Leek and Storey and Friguet et al. studied multiple testing under the factor models [4, 17, 18]. For a general class of dependent models, Leek and Storey, Friguet et al*.*, Fan et al*.*, and Fan and Han showed that overall dependence could be very weakened by reducing the common factors. Modified *P*-values can be used to build more powerful FDR methods [17–20]. The studies by Hall and Jin, and Li and Zhong showed that multiple testing and covariance structures can be used through conversion to make the test statistic, and the results indicated the beneficial effects of dependence [21–23].

However, the above methods rely heavily on the accuracy of estimated models and the asymptotic assumptions of the test statistics. Under small sample conditions, poor estimates of model parameters or violation of independence hypotheses may lead to less powerful or invalid FDR methods. Risser developed a theoretical approach of Bayesian decision for multiple dependent tests and a nonparametric hierarchical statistical model, which controls the FDR and is a strong model for determining the false model. Du et al*.* created a class of multiple testing without distribution for controlling the FDR under general dependency by considering a sequence of symmetric ranking statistics [20, 21].

In many cases, especially in high-dimensional data, consecutive test statistics have a moderate or strong correlation [24–26]. Although in high-dimensional and fused high-low order biological information some techniques such as machine learning or graph representation learnings are developed to handle the complex structures between features, feature selection by MCPs before using these techniques could improve their results [27–29].

Benjamini and Tille and Clark and Hall argued that the state of dependency in the multiple testing is asymptotic with the same independence [10, 11]. But, general dependency structures in multiple testing is still a very challenging and important problem. Efron noted that solidarity should be considered in deciding whether zero hypotheses are important because the accuracy of FDR techniques is compromised in high-correlation situations [9]. However, even if procedures are valid under specific dependency structures, regardless of real dependency information, they will continue to suffer from reduced performance.

Due to the widespread use of the BH procedure, considering the effect of correlation in practical analysis is important. Previous studies evaluated two type of correlation structures; correlation among features and correlated samples. The studies by Storey et al., Hall and Jin, Sun and Cai, and Li and Zhong focused on correlated samples [1, 10, 19]. In the present study, we consider the correlation between features that leads to dependent test statistics, so to modify the BH procedure we accommodate the correlation between sorted features based on the absolute values of corresponded test statistics. For correct inference, this study modified the FDR procedure according to an arbitrary correlation structure and proposed three modified procedures based on conditional fisher information of consecutive sorted test statistics for controlling the false discovery rate.

In the present study, we proposed three modifications to the FDR procedure which can counteract the correlation between sorted features based on the conditional fisher information between consecutive sorted test statistics, and applied them for high dimensional hypothesis testing. Our proposed methods suggested for simultaneous hypothesis testing in two major groups;

1. For simultaneous comparison of P features in two groups; Such as genomic data of a specific disease we have thousands of features for two groups (case/control), so we must have done P hypothesis testing to find the feature(s) with significant difference between groups.
2. For pairwise comparison of a unique feature among k independent groups;Such as Post Hoc tests after ANOVA, we must have done k(k-1)/2 hypothesis testing to find the group(s) with significant differences.

The correlation structure between test statistics in both categories are exist and obviously, it is not ignorable. We applied our modified procedures for first category of simultaneous high dimensional hypothesis testing but it could be applied simply for the second category.

## Results

### Results of the simulation study

Table 1 compares the mean and the standard deviation (SD) of the number of screened features without adjustment on the *p*-values, and with adjustment by Bonferroni (BF), Benjamini–Hochberg (BH), Benjamini–Yekutieli (BY), and three proposed modified procedures under mild (M3), moderate (M2), and strong (M1) assumptions, according to the level of the correlation coefficient (ρ = 0, 0.2, 0.4, 0.5, 0.6, 0.8, 0.9, 0.95, 0.99) between consecutive sorted test statistics by their *p*-values.

**Table 1:** The mean and the standard deviation (SD) of the number of screened features by BF, BH, BY, M1, M2, and M3 procedures in the simulation study

ρ		Adjustment Procedures						
		Non	BF	BH	BY	M1	M2	M3
0	Mean	353.351	49.357	102.112	228.404	214.429	220.73	227.456
	SD	11.801570	5.802294	11.00773	14.75712	14.25881	14.49086	15.70872
0.2	Mean	352.212	48.633	226.385	101.588	222.139	207.094	193.784
	SD	19.659031	9.4009941	26.035332	19.48784	25.869149	25.129637	24.196628
0.4	Mean	352.297	48.607	225.246	100.605	209.667	184.369	162.728
	SD	33.779963	16.182349	45.693253	33.99483	44.655372	42.601335	40.780916
0.5	Mean	351.997	48.567	225.095	100.674	201.033	171.72	147.963
	SD	41.922673	19.446019	56.70119	41.75307	54.320462	51.319834	48.004584
0.6	Mean	352.626	48.713	223.98	100.679	188.669	157.807	133.011
	SD	48.744478	23.358516	65.778312	49.88433	62.063107	58.015391	54.002399
0.8	Mean	351.314	48.654	222.664	100.403	153.56	124.238	101.873
	SD	64.601368	31.025713	86.84043	64.95349	74.744586	67.477378	60.448514
0.9	Mean	351.197	48.334	221.729	100.706	126.631	101.953	83.708
	SD	73.050329	34.773778	97.059054	73.27475	74.665131	66.30559	58.186806
0.95	Mean	350.85	48.39	220.743	100.607	106.651	86.614	72.685
	SD	76.59104	36.74164	102.0592	76.72027	68.18917	60.23118	53.29142
0.99	Mean	351.06	48.43	220.587	101.008	82.937	70.063	61.414
	SD	80.011926	38.365414	106.24165	79.94535	52.212615	47.445633	43.757392

When the correlation coefficient between all features is zero the number of features with *p*-value less than 0.05 have the mean and the standard deviation equal to 353.35, and 11.80, respectively. Also, the mean number of screened features by BH procedure is approximately equal with all three modified procedures M1, M2, and M3. However the mean number of screen features by BY procedure is considerably less than all other procedures except BF. The mean number of screen features by BY procedure reaches to M1, M2, and M3 procedures when the correlations are 0.95, 0.9, and 0.8, respectively. It means that under high levels of correlations the BY procedures are performed approximately equal to the modified procedure. As shown in Table 1, with an increase in correlation coefficients the mean number of screened features without adjustment on their *P*-values are approximately constant, but the standard deviations increased by ρ. This pattern exists for BF, BY, and BH adjustment procedures. But for the M1, M2, and M3 procedures both means and standard deviations have changed according to the correlation between features. The mean number of the screened features decreased according to the increase in the level of correlations in the three proposed methods, but their standard deviations increased.

**Fig. 1:**
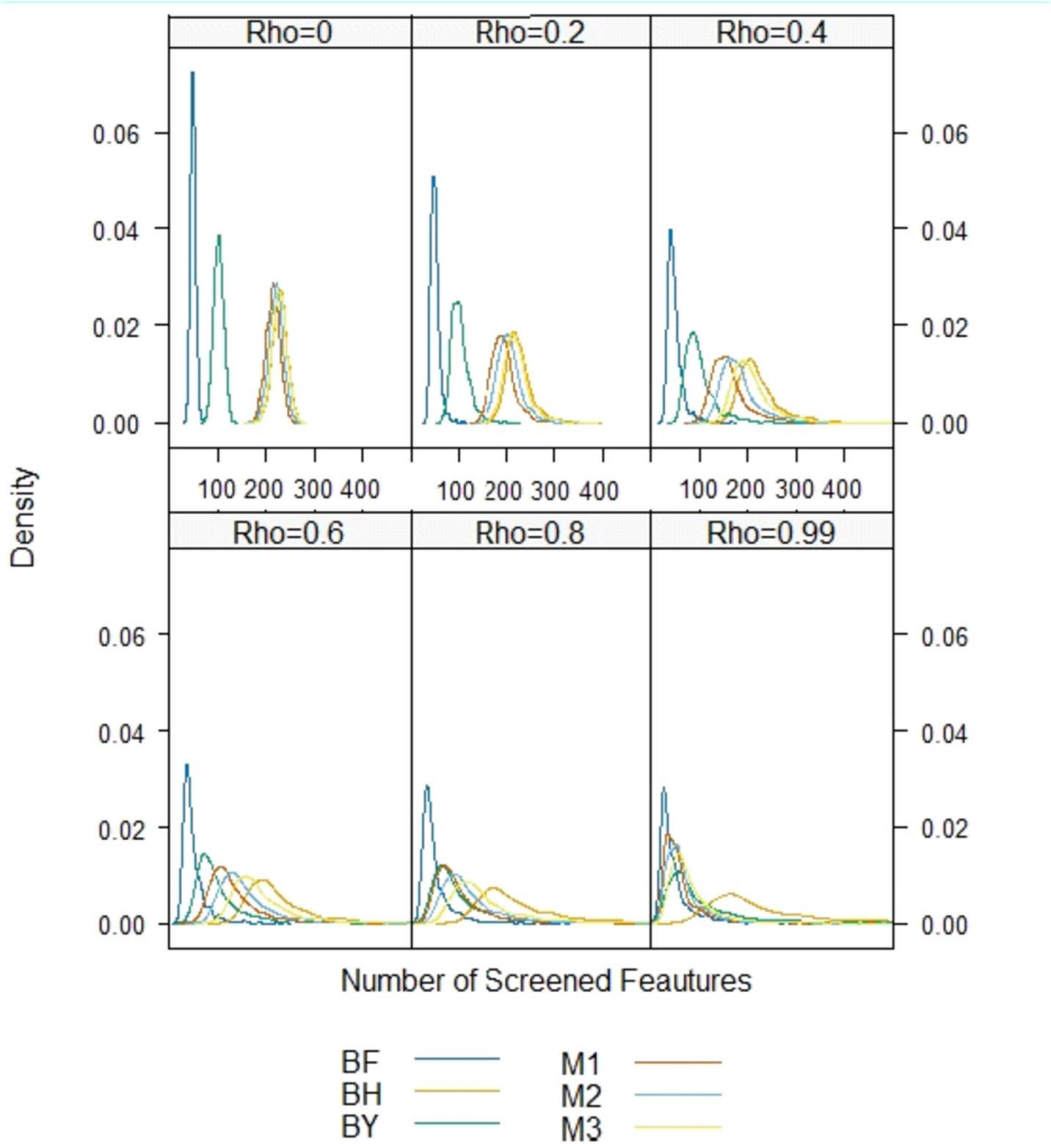
Density plot of the number of discoveries by MCP procedures at the different level of correlations in simulation study

**Fig. 2:**
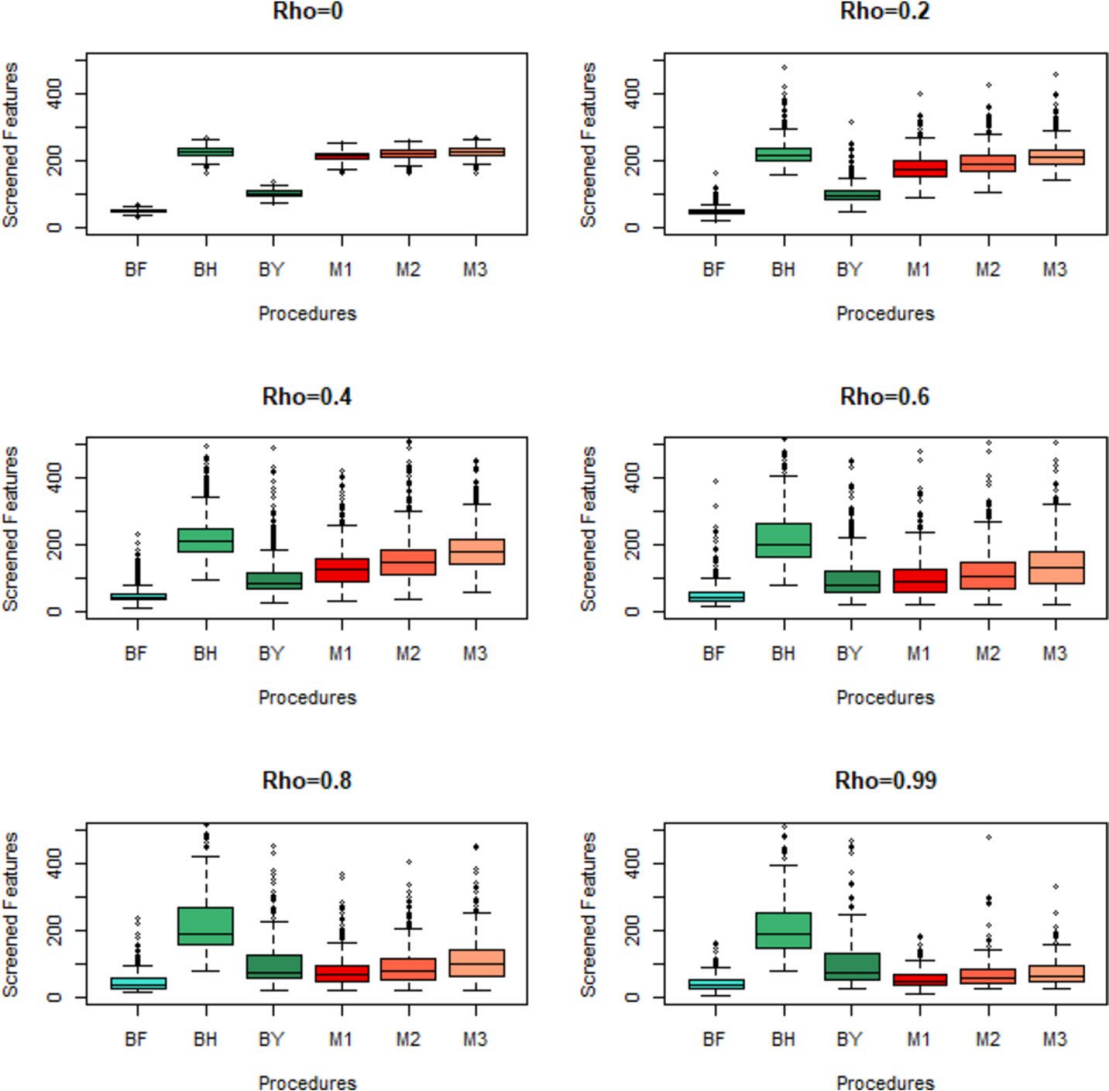
Box plots of the number of discoveries by MCP procedures at different level of correlations in simulation study

As expected the number of screened features by the M3 procedure is less than the number of screened features by the moderate modification M2. The number of screened features by M2 is less than the number of screened features by the mild modification M3. The standard deviations of the number of the screened features increase with the level of correlation in all proposed procedures. As shown in Fig. 1. when $\rho =0$ the distribution of the number of screen features are symmetric, but the kurtosis of BF and BY procedures are higher than normal density. The distribution of screen features by BH, M1, M2, and M3 procedures are approximately identical and normal. By increasing $\rho$ distribution of screen features by all procedures is skewed to right and the skewness increases with$\rho$. The box plots of the number of screened features to compare the median, Interquartile Range (IQR), and outliers are presented in Fig. 2. From these plots, we can observe that despite the increase in outliers, with the increase in, the IQR of modified procedures is smaller than BH and BY procedures. The range distance between the first and the third quartiles with the median for modified procedures is approximately equal and symmetric in comparison to BY and BH procedures. More descriptive statistics of screen features in simulation study are presented in Additional file 1: S1. Also, the results of the othersimulation study when the sample sizes at each group are equal to 30, are presented in Additional file 2: S2.

### Results of the real study

Based on the *p*-values of the t-tests, on P = 22,277 gene expressions and at the level of the α = 0.05, 8465 gene expressions were significant but, most of these genes are not involved in cancer. Since α, type I error rate was not reported in this study, we first determined the power (1-β) based on the different values of the effect size and type I error rates, using the following formula,

$$1-\beta =2\times \left\{1-\varphi \left(\delta \sqrt{n/2}-{Z}_{1-\alpha /2}\right)\right\},$$

where φ, is the cumulative Gaussian density function, n = 55, the sample size in the healthy tissue group, δ = 0.75 is the midpoint between 0.5 to 1, or between the moderate to large effect size, and α = 5 × 10^–12^, and 5 × 10^–10^. So the calculated powers for individual tests are 1-β = 98.9%, and 99.9%, respectively.

Due to the high-dimension data, when performing this hypothesis test, the main concern is to keeping the trade between control the amount of type 1 error (i.e., to keep the family-wise type I error rate at its nominal level α, such as BF procedure) and the power of the study to screening the significance features, by using the FDR procedures to screen the more relevant gene expressions to colorectal cancer. We compared the performance of BH, BY and three proposed modified procedures; M1, M2, and M3 in Table 2.

**Table 2:** Cross tab of the *p*-values and Pearson correlation coefficients between sorted features of colorectal cancer study

*P*-value Categories	n %within columns	Correlation Categories									
		− .08, -0.6	− 0.6, −0.4	− 0.4, −0.2	− 0.2, 0.0	0.0, + 0.2	+ 0.2, + 0.4	+ 0.4, + 0.6	+ 0.6, + 0.8	+ 0.8, + 1.0	Total
0.000, 5 × 10^–12^	n	17	15	0	1	0	0	8	55	12	108
	%	12.6	1.6	0.0	0.0	0.0	0.0	0.6	15.0	75.0	0.5
5 × 10^–12^, 5 × 10^–10^	n	13	40	13	2	0	7	36	33	0	144
	%	9.6	4.1	0.4	0.0	0.0	0.2	2.7	9.0	0.0	0.6
5 × 10^–10^, 5 × 10^–8^	n	14	103	67	4	6	59	103	25	0	381
	%	10.4	10.7	2.1	0.1	0.1	1.7	7.7	6.8	0.0	1.7
5 × 10^–8^, 5 × 10^–4^	n	33	234	502	426	437	559	280	58	1	2530
	%	24.4	24.2	16.1	6.5	6.9	16.0	20.9	15.8	6.3	11.4
5 × 10^–4^, 5 × 10^–2^	n	21	195	757	1650	1561	813	254	50	1	5302
	%	15.6	20.2	24.2	25.3	24.7	23.2	19.0	13.7	6.3	23.8
5 × 10^–2^, 1 × 10^–1^	n	2	47	222	566	550	234	59	15	1	1696
	%	1.5	4.9	7.1	8.7	8.7	6.7	4.4	4.1	6.3	7.6
1 × 10^–1^, 1.000	n	35	333	1564	3861	3762	1830	600	130	1	12,116
	%	25.9	34.4	50.0	59.3	59.6	52.3	44.8	35.5	6.3	54.4
Total		135	967	3125	6510	6316	3502	1340	366	16	22,277

**Fig. 3:**
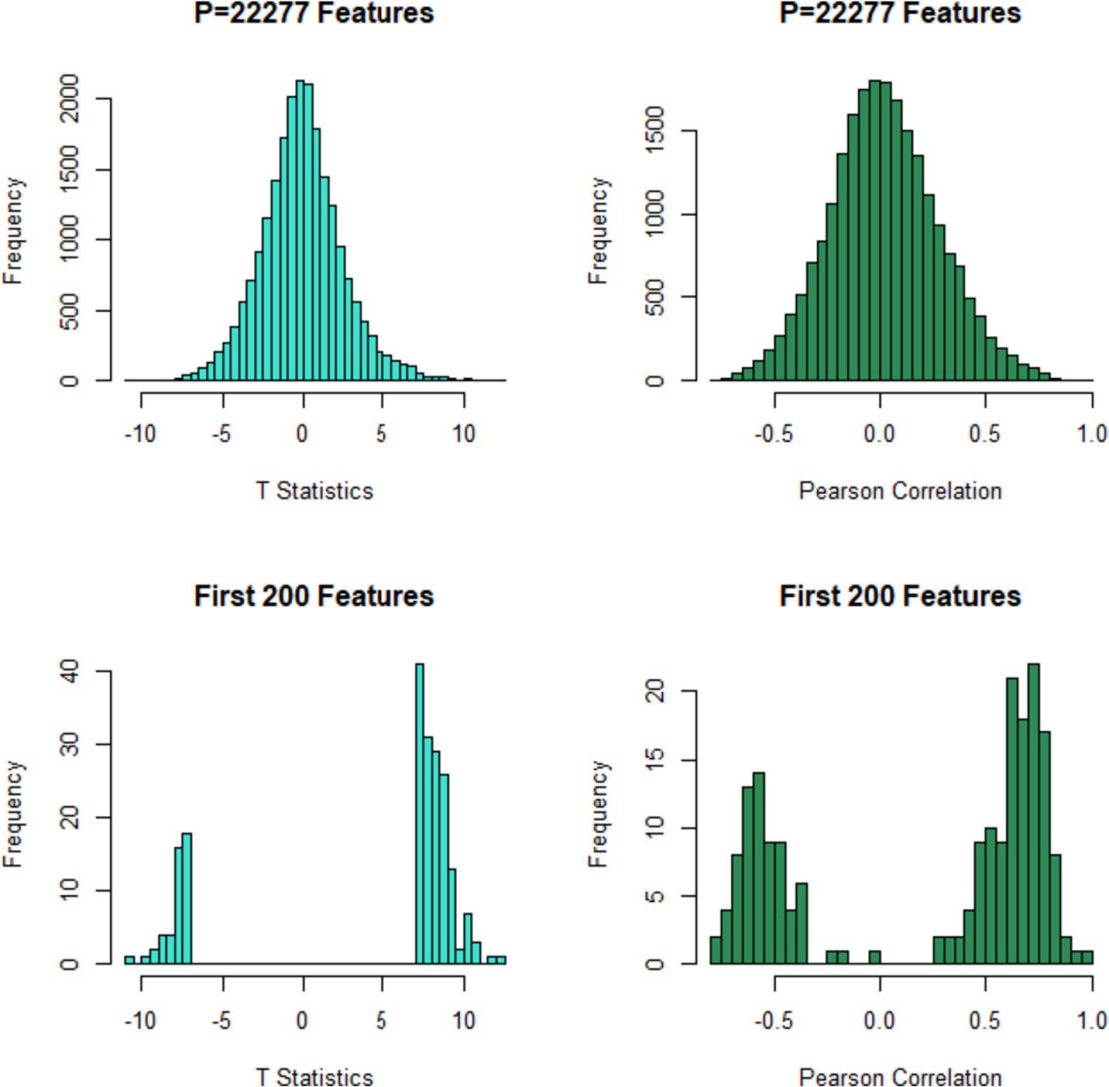
Histogram of t-values and bivariate correlations between sorted features of colorectal gene expression data

Firstly, we show the distribution of the 22,277 t-values and bivariate correlations between sorted features by their *p*-values in Fig. 3. As shown in these histograms the distribution of t-values and correlations are symmetric around zero. For more exploration we draw and show the distribution of the first 200 t-values and bivariate correlations between sorted features by their *p*-values in Fig. 3. As expected the t statistics for first 200 t-values have bi-module distribution on two tailed of the histogram of t-values for 22,277 features. However, the histogram of correlations is more exciting. The correlations between first 200 features are high and also has bi-module distribution on the taileds of the histogram of bivariate correlations between 22,277 consecutive sorted gene expressions. So, independence assumption in the BH procedure is violated and it is necessary to consider the correlation structure in the FDR procedures.

**Fig. 4:**
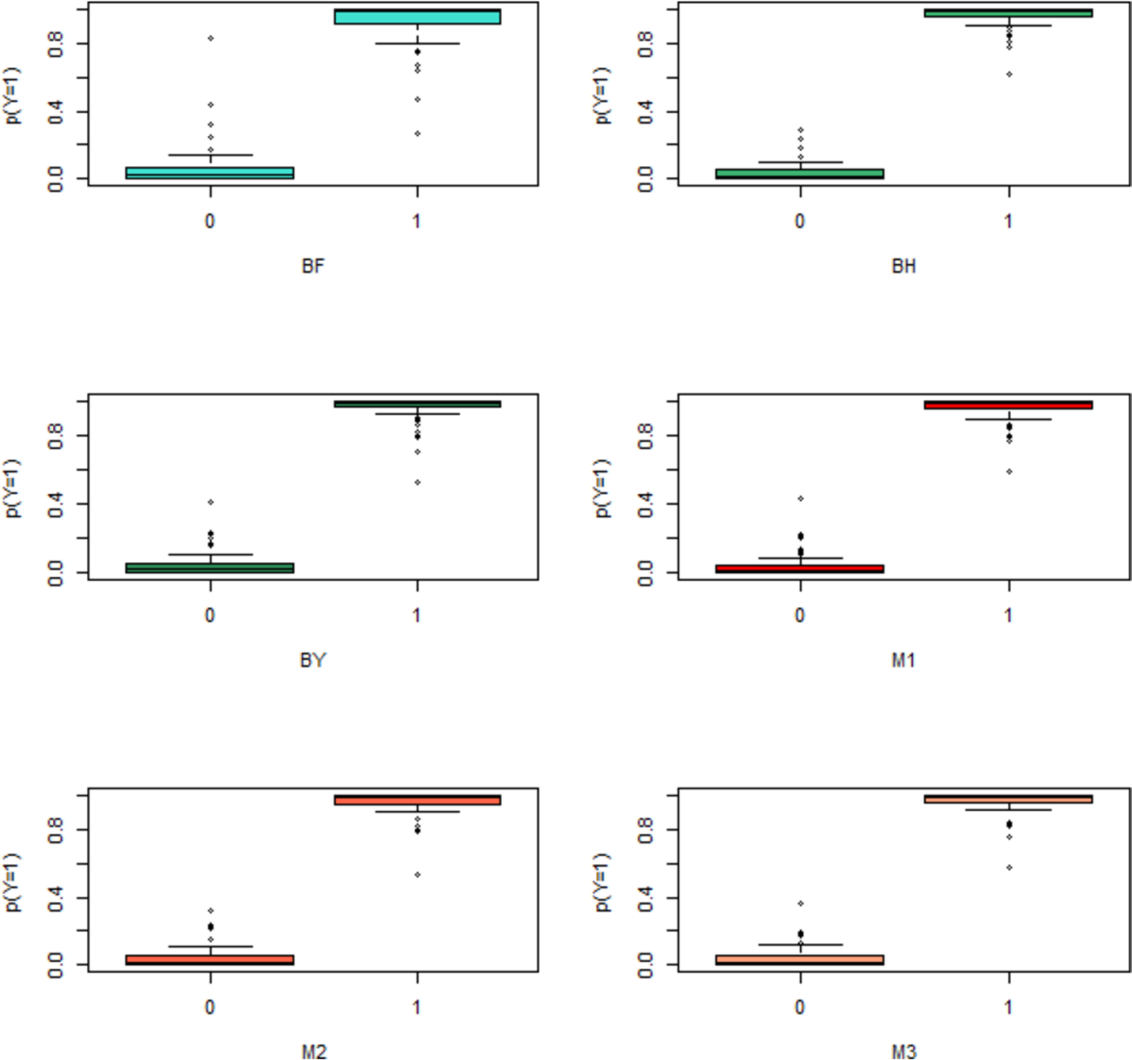
Box plots of predicted probability for Y = 1 versus observed value of Y, from fitted EBLR model on the screen genes by different MCP procedures for colorectal cancer data

Table 3 shows the number of screened features by six adjustment procedures at two levels of α. Also the entropy and AUC for the EBLR models were reported in this table. As shown in Table 3 the number of screened features by BF and BY procedures are equal. Also, the number of screen features by M1 and M2 procedures are equal. The numbers of screen feature at α = 5 × 10^–12^, by all six procedures are few and the Entropies are approximately equal. So, we prefer to use α = 5 × 10^–10^, which gains more power and compares the performance of FDR procedures at this level of type I error rate. At this level of α, the number of screened features increased considerably for all adjustment procedures. Also, there is a considerable difference in screen features by the different adjustment procedures. By fitting the EBLR model on screened features by six adjustment procedure, the entropies and the AUCs were calculated. As seen in this table all the AUCs are 1 (perfect fit) except for the BF procedure. The entropies are near together, but the entropy of the EBLR model on 94 screen features by the BH procedure is equal to 0.82, and the entropy of the EBLR model on 61 screen features by the M1 procedure is equal to 1.19, it means that with losing 94–61 = 33 degree of freedom the reduction in entropy is just equal to 1.19–0.82 = 0.37, so the efficiency of M1 procedure is more than the BH procedure. Also the difference between the entropy of the EBLR models fitted on screened features by M2 and M1 procedures is ignorable in compare with loss in degree of freedoms. The box plots in Fig. 4, show that the predicted probability of the EBLR model completely separated in cancerous and healthy tissue for M1, M2, M3, and BY procedures, but for BF and BH procedures there is no complete separation. Although, the box plot of the BY procedure shows perfect fit the entropy of the EBLR model fitted on 61 features from the M1 procedure, and 71 features from the M2 procedure, and 81 features from the M3 procedure are less than the entropy of the EBLR model fitted on the 59 screen features by BY procedure. So, the M1, M2 and M3 procedures are more efficient than BY procedure. So, finally the M1 procedure with 61 screened features is the most efficient procedure for feature screening in colorectal cancer data according to less entropy with less loss in degree of freedom.

**Table 3:** The entropy, and the Area Under the ROC Curve (AUC) of fitted EBLR models on the screened genes by BF, BH, PY, M1, M2, and M3 procedures of colorectal cancer study

$\alpha$	Indices	Without Adjustment	BF	BH	BY	Modified Procedures		
						M1	M2	M3
5 × 10^–12^	K	108	12	24	12	14	14	17
	Entropy	0.439	25.256	16.981	25.256	20.988	20.988	20.203
	AUC	1.000	0.968	0.986	0.968	0.976	0.976	0.979
5 × 10^–10^	K	252	37	94	59	61	71	81
	Entropy	0.094	8.097	0.823	1.716	1.193	1.407	1.120
	AUC	1.000	0.996	1.000	1.000	1.000	1.000	1.000

## Discussion

The BH procedure for feature screening based on controlling the false discovery rate has a substantial assumption of independent test statistics. In large-scale multiple testing assumption of independence between test statistics is unrealistic. Many studies reported that the dependency structure between test statistics cause over-dispersion in the distribution of the FDR [4–8]. In the present study, we observe that the over-dispersion and right skewness in the distribution of the number of screen features by BF, BH, BY and proposed procedures increase with the level of correlations. However, as shown in Fig. 1 the skewness in the density of the proposed procedures is less than the BH procedure, and also the interquartile range of boxplots in Fig. 2 was thinner than BH and BY procedures.

When there was no correlation between 1000 simulated features, the performance of the BH procedure was similar to the three proposed procedures, but the BY procedure is very conservative as reported [4]. In low to medium correlation structures the BY procedure is too conservative, and the BH procedure is too liberal. The mean number of screened features by BH and BY procedures were constant at the different level of the correlation between features. The mean number of screen features by our proposed procedures were between BY and BH procedures and reduced when the level of correlations increased. Where the correlations between features were high (ρ > 0.8) the number of screened features by proposed procedures reach to the BF procedure, as expected. We reduced the acceleration of increasing the number of false discoveries by modifying the BH procedure according to the amount of extra information of each new feature, resulting in a more precise procedure for screening the important features with the presence of a solidarity structure between the features.

Then, we compared the performance of three proposed procedures with BF, BH and BY for screening in High-dimensional genomic dataset, with 22,277 gene expressions' comparisons between the healthy and cancerous tissue groups. In this regard, by allowing two different levels for nominal type I error rates, α, the significance genes were screen by six procedures. The Efficient Bayesian Logistic Regression (EBLR) model were used to fit a predictive model based on the screened features. The EBLR model based on the screen features by M1 and M2 procedures have minimum entropies and were more efficient than BY and BH procedures. In a study on this data set twenty Machin Learning approaches were used to fit the predictive model based on the screened features. The maximum AUC was 0.94 obtained by Deep Neural Network (DNN) and Logistic Model Tree (LMT) [27].

Leek and Storey developed an approach to address the strong arbitrary dependence of multiple testing collected on the original data surface in a large-scale (high-dimensional data) study before calculating the test statistics or *P*-values. To address the dependency problem of multiple testing based on kernel dependency estimation, they presented a small set of vectors that define entirely the dependency structure in any high-power data set. They showed that hypothesis tests could be randomly independent as long as conditioning on a dependence kernel. This generalizes the results of the independent error rate control to the general dependency mode. It can also estimate dependence at the data level, which is more useful than estimating dependence at the *P*-value level or test statistics [23]. Compared with proposed procedures this method is blind and base on the random correlation structures, but our modifications are based on the ordered information of whole data set.

Although, some efficient methods for the low to high-correlated feature have been proposed and used, our proposed procedures are the first to modify the thresholds of the FDR procedure based on the information theory. So, according to the results of the simulation study and real data study, the optimization in the number of screened features has occurred.

## Conclusion

The modified proposed procedures based on information theory, are much more flexible than BH and BY procedures for the amount of correlation between test statistics. Our modified procedures avoided screening the non-informative features and so the number of screened features reduced with the increase in the level of correlation.

The three proposed modified procedures for feature screening are simply applicable for arbitrary positive or negative, and low or high correlation structures between sorted test statistics. These modifications are based on information theory and lead to finding the small set of significant features with sufficient information according to correlation between the sorted features and so, the remaining features do not have extra information.

## Methods

First, we describe the Benjamini–Hochberg (BH) procedure and Benjamini–Yekutieli (BY) procedure then introduce our proposed modified procedures.

### Benjamini–Hochberg procedure (BH Procedure)

In this procedure, when test statistics under the distribution of the null hypothesis are independent, the BH procedure control the FDR at the level of α. The BH procedure is shown below:

1. Sorting the observed *p*-values in ascending order, ${p}_{(1)}\le \dots \le {p}_{(P)}$

2. Calculation of $k={\text{max}}\{1\le {\text{i}}\le {\text{P}}:{p}_{\left(i\right)}\le \frac{{l}_{i}}{{\text{P}}}\alpha \}$ where ${l}_{i}=i\quad for \quad i=\mathrm{1,2},\dots ,P$

3. If there is such a K, all the null hypotheses corresponding to ${p}_{(1)}\le \dots \le {p}_{\left(k\right)}$ are rejected.

### Benjamini–Yekutieli procedure (BY procedure)

The Benjamini–Yekutieli proposed a procedure for controling the false discovery rate under arbitrary dependency (test statistics have positive or negative correlations). They modified the threshold of BF procedure using a constant function $C\left(P\right)=\sum_{i=1}^{P}\frac{1}{i}$. And find.

$$k={\text{max}}\left\{1\le {\text{i}}\le {\text{P}}:\quad{p}_{\left(i\right)}\le \frac{{l}_{i}}{{\text{P}}\times {\text{C}}\left({\text{P}}\right)}\alpha \right\}.$$

But in situation that the tests statistics are independent or positively correlated they suggested C(P) = 1 like as an ordinary BH procedure.

### Proposed modified procedures

Consider the simultaneously P hypotheses:

1 $$\left\{\begin{array}{l}{{\text{H}}}_{0{\text{i}}} : {\delta }_{i}=0 \\ \\ {{\text{H}}}_{1{\text{i}}} : {\delta }_{i}\ne 0\end{array}\right.for\quad i= 1, 2, \dots .,P.$$

where ${\delta }_{i}=\left|{\mu }_{1i}-{\mu }_{2i}\right|$, is the absolute mean difference between two groups of the ith feature; ${\mu }_{1i}, is\; the\; mean\; of\; the\; ith\; feature\; at\; the\; first\; \left(case\right) group.$ ${\mu }_{2i}, is\; the\; mean\; of\; the\; ith\; feature\; at\; the\; second\; \left(control\right) group.$

If we assume that all features are independent and following the multivariate Gaussian distribution with mean ${\varvec{\delta}}=({\delta }_{1},{\delta }_{2},\dots ,{\delta }_{P})$ and diagonal covariance matrix **Σ.**

We could scaled each ${\delta }_{i}s$ by dividing on their variances:

$${\tau }_{i}=\frac{{\delta }_{i}}{\left(\sqrt{\frac{{\sigma }_{1i}^{2}}{{n}_{1}}+\frac{{\sigma }_{2i}^{2}}{{n}_{2}}}\right)},$$

where; $${\sigma }_{1i}^{2}, is\; the\; variance\; of\; the\; {i}{th}\; feature\; at\; the\; first\; \left(case\right) group.$$ $${\sigma }_{2i}^{2}, is\; the\; variance\; of\; the\; {i}{th}\; feature\; at\; the\; second\; \left(control\right) group.$$ $${n}_{1} \& {n}_{2}, are\; the \; sample \; sizes \; of \; the \; first \; and \; second\; groups, \; respectively.$$

So we rewrite the hypotheses (1) as follow:

2 $$\left\{\begin{array}{l}{{\text{H}}}_{0{\text{i}}} : {\tau }_{i}=0 \\ \\ {{\text{H}}}_{1{\text{i}}} : {\tau }_{i}\ne 0\end{array}\right.for\quad i= 1, 2, \dots .,P.$$

The t-test statistics for (2) is as follows:

$${t}_{i}=\frac{\left|{\overline{X} }_{1i}-{\overline{X} }_{2i}\right|}{{S}_{i}\sqrt{\frac{1}{{n}_{1}}+\frac{1}{{n}_{2}}} }\quad with\quad d.f={n}_{1}+{n}_{2}-2.$$

where ${\overline{X} }_{1i}, is\; the\; sample\; mean\; of\; the\; {i}{th}\; feature\; at\; the\; first\; \left(case\right)\; group,$ ${\overline{X} }_{2i}, is\; the\; mean\; of\; the\; {i}{th}\; feature\; at\; the\; second\; \left(control\right)\; group,$ ${S}_{1i}^{2},\; is\; the\; sample\; variance\; of \; the\; {i}{th}\; feature\; at \; the\; first\; \left(case\right) \; group,$ ${S}_{2i}^{2}, is\; the\; sample\; variance\; of\; the\; {i}{th}\; feature\; at\; the\; second\; \left(control\right)\; group,$ ${S}_{i}= \sqrt{\frac{\left({n}_{1}-1\right){S}_{1i}^{2}+{(n}_{2}-1){S}_{2i}^{2}}{{n}_{1}+{n}_{2}-2}} , is\; pooled\; variance\; of\; the\; {i}{th}\; feature\; in\; both\; groups,$ ${n}_{1}, {n}_{2},\; are\; the\; sample\; sizes\; of\; the\; first\; and\; second\; groups,\; respectively,$ if n_1_ and n_2_ are large enough,$\left({n}_{1}+{n}_{2}-2\right)\ge 30$, ${t}_{i} s$ follows Gaussian distribution with mean ${\varvec{\tau}}=({\tau }_{1},{\tau }_{2},\dots ,{\tau }_{P})$ and covariance matrix I. So we use Z_i_ instead of t_i_.

According to information theory when $\left|{\overline{X} }_{1i}-{\overline{X} }_{2i}\right| s$ are independent multivariate Gussian random variables, the fisher information of ${\delta }_{i}$, conditional on ${\delta }_{i-1}$ is as follow;

$${I}_{({\delta }_{i}\left|{\delta }_{i-1}\right)}\left(\left({\overline{X} }_{1\left(i\right)}-{\overline{X} }_{2\left(i\right)}\right)\mid\left({\overline{X} }_{1\left(i-1\right)}-{\overline{X} }_{2\left(i-1\right)}\right)\right)=1/{\sigma }_{i}^{2}$$

Also, ${Z}_{i} s$ are independent multivariate standard Gaussian random variables, so the fisher information of ${\tau }_{i}$, conditional on ${\tau }_{i-1},$ is ${I}_{({\tau }_{i}\left|{\tau }_{i-1}\right)}({Z}_{i}\left|{Z}_{i-1}\right)=1,\, for\, i=2, 3,$ …, P. Also the fisher information of P independent Gaussian features is equal to I(${\tau }_{1},{\tau }_{2}, \dots , {\tau }_{P})=P$. So,

$${I}_{({\tau }_{i}\left|{\tau }_{i-1}\right)}({Z}_{\left(i\right)}|{Z}_{(i-1)})=\frac{1}{P} {I}_{({\tau }_{1},{\tau }_{2}, \dots , {\tau }_{P})}({Z}_{(1)},{Z}_{(2)}, \dots , {Z}_{(P)})$$

In BH procedure according to independence assumption, the step-up conditional thresholds increase by $\frac{1}{P}$. But when the features are correlated, if $Corr\left({X}_{(i)},{X}_{(i-1)}\right)={\rho }_{i}$ we have:

$$Corr\left({\overline{X} }_{1(i)}-{\overline{X} }_{2(i)},{\overline{X} }_{1(i-1)}-{\overline{X} }_{2(i-1)}\right)=Corr\left({Z}_{\left(i\right)},{Z}_{(i-1)}\right)={\rho }_{i}.$$

So, the fisher information of ${\delta }_{i}$, conditional on ${\delta }_{i-1}, i\ne j,$ is as follow,

$${I}_{({\delta }_{i}\left|{\delta }_{i-1}\right)}\left(\left|{\overline{X} }_{1i}-{\overline{X} }_{2i}\right|\mid\left|{\overline{X} }_{1(i-1)}-{\overline{X} }_{2(i-1)}\right|\right)=\left(1-{\rho }_{i}^{2}\right)/{\sigma }_{i}^{2}$$

And the fisher information of ${\tau }_{i}$, conditional on ${\tau }_{i-1}, i\ne j,$ is as follow:

3 $$\dddot I_{{(\tau_{i} {|}\tau_{i - 1} {)}}} \left( {Z_{\left( i \right)} |Z_{{\left( {i - 1} \right)}} } \right) = \left( {1 - \rho_{i}^{2} } \right)$$

So, under mild condition we propose the conditional thresholds increase by (3).

As $\left(1-{\rho }_{i}^{2}\right)\le 1$ the information of ${\tau }_{i}$, conditional on ${\tau }_{i-1}$ decrees when both variables are correlated. It is clear, because when two variables are correlated, a part of information of the second variable is defined in the first variable. As $Corr\left({Z}_{\left(i\right)},{Z}_{(i-1)}\right)={\rho }_{i}$ we could define two independent consecutive sorted standardized Gaussian test statistics as

$$Z^{\prime}_{{\left( {i - 1} \right)}} = Z_{{\left( {i - 1} \right)}} \quad Z^{\prime}_{\left( i \right)} = \frac{{ - \rho_{i} }}{{\sqrt {1 - \rho_{i}^{2} } }}Z_{{\left( {i - 1} \right)}} + \frac{1}{{\sqrt {1 - \rho_{i}^{2} } }} Z_{\left( i \right)}$$

In genomic high dimensional datasets, features are measured for unique source (patient) so we could have a strong assumption that all effects (absolute mean differences) are identical with Gaussian distribution, but the correlation between features are different. As a Result

$$Z^{\prime}_{\left( i \right)} = \left( {\frac{{ - \rho_{i} }}{{\sqrt {1 - \rho_{i}^{2} } }} + \frac{1}{{\sqrt {1 - \rho_{i}^{2} } }}} \right) Z_{\left( i \right)}\, \to\, \sigma_{{Z_{\left( i \right)} }}^{2} = \frac{{1 - \rho_{i}^{2} }}{{\left( {1 - \left| {\rho_{i} } \right|} \right)^{2} }} = \frac{{1 + \left| {\rho_{i} } \right|}}{{1 - \left| {\rho_{i} } \right|}}$$

So, the conditional fisher information under strong assumption is

4 $${\dot{I}}_{({\tau }_{i}\left|{\tau }_{i-1}\right)}\left({Z}_{\left(i\right)}|{Z}_{\left(i-1\right)}\right)=\frac{1}{{\sigma }_{{Z}_{\left(i\right)}}^{2}}=\frac{1-\left|{\rho }_{i}\right|}{1+\left|{\rho }_{i}\right|}$$

So, under strong assumption we propose the conditional thresholds increase by (4).

Also, we can write:

$$\dot{I}_{{(\tau_{i} {|}\tau_{i - 1} {)}}} (Z_{\left( i \right)} |Z_{{\left( {i - 1} \right)}} ) = \frac{{\dddot I_{{(\tau_{i} {|}\tau_{i - 1} {)}}} (Z_{\left( i \right)} |Z_{{\left( {i - 1} \right)}} )}}{{\left( {1 + \left| {\rho_{i} } \right|} \right)^{2} }}$$

AS, $\left(1+\left|{\rho }_{i}\right|\right)\ge 1$, we proposed a moderate modification between strong and mild modification:

5 $$\ddot{I}_{{(\tau_{i} {|}\tau_{i - 1} {)}}} (Z_{\left( i \right)} {|}Z_{{\left( {i - 1} \right)}} {)} = \frac{{\dddot I_{{(\tau_{i} {|}\tau_{i - 1} {)}}} }}{{\left( {1 + \left| {\rho_{i} } \right|} \right)}} = \left( {1 - \left| {\rho_{i} } \right|} \right)$$

As a result, under moderate condition we propose the conditional thresholds increase by (5).

The step-down procedure works after sorting absolute values of Z_i_, in descending order. Supposed that Z_(i)_ is the ith sorted test statistics and $Corr\left({Z}_{\left(i-1\right)},{Z}_{\left(i\right)}\right)={\rho }_{i}$ for i = 2,3,…,P.

In case of ${\rho }_{i}\ne 0$, the FDR procedure should be modified based on this correlation coefficient. The Pearson correlation coefficient r_i_, as an estimator of ${\rho }_{i}$, between sorted consecutive features according to their *p*-values was used to the modifications on the FDR procedure.

We propose three (strong, moderate, and mild) modifications on the threshold of the BH procedure. So, the thresholds, l_i_, based on the conditional Fisher information under mild, moderate, and strong assumptions were suggested as follow:

1. Mild modification, where $\dddot l_{i} = \dddot l_{i - 1} + \left( {1 - \left| {r_{i} } \right|^{2} } \right)$
2. Moderate modification, where ${{\ddot{l}}_{i}=\ddot{l}}_{i-1}+\left(1-\left|{r}_{i}\right|\right)$
3. Strong modification, where ${{\dot{l}}_{i}=\dot{l}}_{i-1}+\left[\frac{\left(1-\left|{r}_{i}\right|\right)}{\left(1+\left|{r}_{i}\right|\right)}\right]$

For $i=1\dots \dots P$, and we define $\dot{l}_{1} = \ddot{l}_{1} = \dddot l_{1} = 1$.

So, our procedures work as follow,

1. Sorting the observed *P*-values in ascending order, ${p}_{(1)}\le \dots \le {p}_{(P)}$
2. Calculating the $Corr\left({X}_{(i)},{X}_{(i-1)}\right)={r}_{i}$, for $i=1, 2, \dots , P$.
3. Calculating the ${l}_{i}s$, for $i = 1, 2, \ldots , P.$
4. Calculation of $$={\text{max}}\{1\le {\text{i}}\le {\text{P}};{p}_{\left(i\right)}\le \frac{{l}_{i}}{{\text{P}}}\alpha \}$$,$$for\, i=\mathrm{1,2},\dots ,P$$.
5. If there is such a K, all the first K sorted *p*-values called significance.

If all the sorted features have a complete linear correlation, we will have

$$if\, \left| {\rho_{i} } \right| = 1 \,\Rightarrow \dot{l}_{{\text{i}}} = \ddot{l}_{{\text{i}}} = \dddot l_{{\text{i}}} = 1\, \forall\, i = 1,2, \ldots , P\, \Rightarrow k = max\left\{ {1 \le i \le P;\,p_{\left( i \right)} \le \frac{\alpha }{P}} \right\}$$

it means that all sorted test statistics have same information in the class of the linear estimation statistics of ${\tau }_{i}$, and so the thresholds of our proposed procedures do not increase for consecutive tests. So, the performance of modified FDR procedures is near to the BF procedure.

If the test statistics are independent the pairwise correlation coefficient between all features are zero, so we have:

It means that, when all sorted test statistics are independent, the performance of three proposed procedures are near to the BH procedure.

We compared the adjusted thresholds and the adjusted *p*-values procedures of BF, BH, BY and three proposed procedures; strong (M1), moderate (M2), and mild (M3) by the rank of the sorted *p*-values in Table 4. Except for the BY procedure, the first *p*-value compared with $\frac{1}{P}\alpha$ in all other procedures. The thresholds of BF procedure are fixed and there is no increase with the rank of the sorted *p*-values. Both BH and BY thresholds increased constantly by the rank of the sorted *p*-values, $\frac{k}{P}\alpha$ and $\frac{k}{P\times C(P)}\alpha$, respectively. The thresholds of M1, M2, and M3, increased by the rank of sorted *p*-values but were proportional to the level of correlation between sorted test statistics. The speed of increases in modified procedures is lower than BH procedure. So, it is expected that the number of screened features by the modified procedures be less than the BH procedure. As $C\left(P\right)>1\, for\, P>1,$ the first threshold of the BY procedure is less than the BF procedure, so, the BY procedure could be more conservative than BF procedure due to its first threshold value.

**Table 4:** Thresholds of BF, BH, BY, M1, M2, and M3 procedures for the sorted *p*-values

Procedures		Rank of *p*-values				
		1	2	3	…	K
I. Adjusted Thresholds	BF	$\frac{1}{P}\alpha$	$\frac{1}{P}\alpha$	$\frac{1}{P}\alpha$	…	$\frac{k}{P}\alpha$
	BH	$\frac{1}{P}\alpha$	$\frac{2}{P}\alpha$	$\frac{3}{P}\alpha$	…	$\frac{k}{P}\alpha$
	BY	$\frac{1}{P\times C(P)}\alpha$	$\frac{2}{P\times C(P)}\alpha$	$\frac{3}{P\times C(P)}\alpha$	…	$\frac{k}{P\times C(P)}\alpha$
	M1	$\frac{1}{P}\alpha$	$\frac{1+\frac{\left(1-\left|{r}_{2}\right|\right)}{\left(1+\left|{r}_{2}\right|\right)}}{P}\alpha$	$\frac{1+(\frac{\left(1-\left|{r}_{2}\right|\right)}{\left(1+\left|{r}_{2}\right|\right)}+\frac{\left(1-\left|{r}_{3}\right|\right)}{\left(1+\left|{r}_{3}\right|\right)})}{P}\alpha$	…	$\frac{1+\sum_{i=2}^{k}\frac{(1-\left|{r}_{i}\right|)}{(1+\left|{r}_{i}\right|)}}{P}\alpha$
	M2	$\frac{1}{P}\alpha$	$\frac{1+(1-\left|{r}_{2}\right|)}{P}\alpha$	$\frac{1+(1-\left|{r}_{2}\right|+1-\left|{r}_{3}\right|)}{P}\alpha$	…	$\frac{1+\sum_{i=2}^{k}(1-\left|{r}_{i}\right|)}{P}\alpha$
	M3	$\frac{1}{P}\alpha$	$\frac{1+(1-{\rho r}_{2}^{2})}{P}\alpha$	$\frac{1+\left(1-{r}_{2}^{2}\right)+(1-{r}_{3}^{2})}{P}\alpha$	…	$\frac{1+\sum_{i=2}^{k}(1-{r}_{i}^{2})}{P}\alpha$
II. Adjusted *p*-values	BF	$P\times$ p_(1)_	$P\times$ p_(2)_	$P\times$ p_(3)_	…	$P\times$ p_(k)_
	BH	$P\times$ p_(1)_	$\frac{P}{2}\times$ p_(2)_	$\frac{P}{3}\times$ p_(3)_	…	$\frac{P}{k}\times$ p_(k)_
	BY	$P\times C\left(P\right)\times$ p_(1)_	$\frac{P\times C(P)}{2}\times$ p_(2)_	$\frac{P\times C(P)}{3}\times$ p_(3)_	…	$\frac{P\times C(P)}{k}\times$ p_(k)_
	M1	$P\times$ p_(1)_	$\frac{P}{1+\frac{\left(1-\left|{r}_{2}\right|\right)}{\left(1+\left|{r}_{2}\right|\right)}}\times$ p_(2)_	$\frac{P}{1+(\frac{\left(1-\left|{r}_{2}\right|\right)}{\left(1+\left|{r}_{2}\right|\right)}+\frac{\left(1-\left|{r}_{3}\right|\right)}{\left(1+\left|{r}_{3}\right|\right)})}\times$ p_(3)_	…	$\frac{P}{1+\sum_{i=2}^{k}\frac{(1-\left|{r}_{i}\right|)}{(1+\left|{r}_{i}\right|)}}\times$ p_(k)_
	M2	$P\times$ p_(1)_	$\frac{P}{1+(1-\left|{r}_{2}\right|)}\times$ p_(2)_	$\frac{P}{1+(1-\left|{r}_{2}\right|+1-\left|{r}_{3}\right|)}\times$ p_(3)_	…	$\frac{P}{1+\sum_{i=2}^{k}(1-\left|{r}_{i}\right|)}\times$ p_(k)_
	M3	$P\times$ p_(1)_	$\frac{P}{1+(1-{\rho r}_{2}^{2})}\times$ p_(2)_	$\frac{P}{P1+\left(1-{r}_{2}^{2}\right)+(1-{r}_{3}^{2})}\times$ p_(3)_	…	$\frac{P}{1+\sum_{i=2}^{k}(1-{r}_{i}^{2})}\times$ p_(k)_

### Illustration example

To demonstrate how we estimate the thresholds and adjusted *p*-values we make an artificial example. Supposed that we did eight individual hypothesis tests to find the significance differences for eight features in two groups and sort their *p*-values as follows,

$$\begin{aligned} {\text{p}}_{{({1})}} & = \, 0.00{23},{\text{ p}}_{{({2})}} = \, 0.00{98},{\text{ p}}_{{({3})}} = \, 0.0{139},{\text{ p}}_{{({4})}} = \, 0.0{221}, \\ {\text{p}}_{{({5})}} & = \, 0.0{348},{\text{ p}}_{{({6})}} = \, 0.0{421},{\text{ p}}_{{({7})}} = \, 0.0{463},{\text{ p}}_{{({8})}} = \, 0.0{52}. \\ \end{aligned}$$

Also we find the Pearson correlation coefficient between two consecutive sorted features by their *p*-values as follow,

$$\begin{aligned} {\text{r2}} & = {\text{ Cor}}\left( {{\text{X}}_{{({2})}} ,{\text{X}}_{{({1})}} } \right) = \, 0.{1},{\text{ r3}} = {\text{ Cor}}\left( {{\text{X}}_{{({3})}} ,{\text{X}}_{{({2})}} } \right) = \, - 0.{5},{\text{ r4}} = {\text{ Cor}}\left( {{\text{X}}_{{({4})}} ,{\text{X}}_{{({3})}} } \right) = \, 0.{9},\\{\text{ r5}} = {\text{ Cor}}\left( {{\text{X}}_{{({5})}} ,{\text{X}}_{{({4})}} } \right) = \, 0.{7}, \, {\text{r6}} & = {\text{ Cor}}\left( {{\text{X}}_{{({6})}} ,{\text{X}}_{{({5})}} } \right) = \, - 0.{8},{\text{ r7}} = {\text{ Cor}}\left( {{\text{X}}_{{({7})}} ,{\text{X}}_{{({6})}} } \right) = \, 0.{2},{\text{ r8}} = {\text{ Cor}}\left( {{\text{X}}_{{({8})}} ,{\text{X}}_{{({7})}} } \right) = 0.{9}. \\ \end{aligned}$$

The purpose of this example is simultaneous comparison of eight features between two groups. So, we must use of an adjustment procedure to control the FDR. We compare the performance of six adjustment procedures to find the simultaneous difference at the significance level of α = 0.1 With two approaches; first, we calculate the adjusted *p*-values and then compared them with α, and secondly, we calculate the adjusted thresholds and compared the sorted *p*-values with them (Table 5). As shown in Table 5, both approaches lead to the same result.

**Table 5:** Adjusted thresholds and adjusted *p*-values by BF, BH, By, M1, M2, and M3 procedures in the illustration example

	Sorted *p*-values	Adjustment Procedures					
		BF	BH	BY	M1	M2	M3
Adjusted thresholds	0.0023	0.0125**	0.0125**	0.0046**	0.0125**	0.0125**	0.0125**
	0.0098	0.0125**	0.0250**	0.0092	0.0227**	0.0238**	0.0249**
	0.0139	0.0125	0.0375**	0.0138	0.0269**	0.0300**	0.0343**
	0.0221	0.0125	0.0500**	0.0184	0.0276**	0.0312**	0.0366**
	0.0348	0.0125	0.0625**	0.0230	0.0298	0.0350**	0.0430**
	0.0421	0.0125	0.0750**	0.0276	0.0311	0.0375	0.0475**
	0.0463	0.0125	0.0875**	0.0322	0.0395	0.0475	0.0595**
	0.0520	0.0125	0.0999**	0.0368	0.0401	0.0488	0.0619**
Adjusted *p*-values	0.0023	0.0184*	0.0184*	0.0500*	0.0184*	0.0184*	0.0184*
	0.0098	0.0784*	0.0392*	0.1065	0.0431*	0.0413*	0.0394*
	0.0139	0.1112	0.0371*	0.1007	0.0517*	0.0463*	0.0406*
	0.0221	0.1768	0.0442*	0.1201	0.0802*	0.0707*	0.0603*
	0.0348	0.2784	0.0557*	0.1513	0.1169	0.0994*	0.0809*
	0.0421	0.3368	0.0561*	0.1526	0.1352	0.1123	0.0886*
	0.0463	0.3704	0.0529*	0.1438	0.1173	0.0975	0.0778*
	0.0520	0.4160	0.0520*	0.1413	0.1296	0.1067	0.0840*

*This adjusted *p*-value is less than α = 0.1

**p_(i)_ is less than the adjusted threshold

### Simulation study

We set the dimension of P = 1000 features in two independent equal groups with size n_1_ = n_2_ = 100 and generate the observations for these features sequentially as the following scheme with 1000 replications.

1. Simulate δ_i_: $${\varvec{\delta}}=\left({\delta }_{1},{\delta }_{2},\dots ,{\delta }_{P}\right)\sim MVNorm\left(0,{\upsigma }^{2}{\varvec{I}}\right)$$ with $${\upsigma }^{2}=0.0678$$
2. Simulate Z_i_:
  $${\text{Zi}}|{\text{group}}=1: {\varvec{Z}}=\left({Z}_{1},{\text{Z}},\dots ,{Z}_{P}\right)\sim MVNorm\left({\varvec{\delta}},(1-\rho ){\varvec{I}}+\rho {\varvec{J}}\right)$$
  $${\text{Zi}}|{\text{group}}=2: {\varvec{Z}}=\left({Z}_{1},{\text{Z}},\dots ,{Z}_{P}\right)\sim MVNorm\left(0,(1-\rho ){\varvec{I}}+\rho {\varvec{J}}\right)$$

with ρ: ρ=0, 0.2, 0.4, 0.5, 0.6, 0.8, 0.9, 0.95, 0.99.

We set the variance of δ equal to 0.0678, to have strong control on the Family Wise Error Rate (FWER). That means we bounded the number of screened features by BF adjustment method less than 5% of the total features regardless of which null hypotheses are true and which are false.

At each replication we conducted P independent sample t-tests and sort their *p*-values. We sort the *p*-values and then calculate the adjusted *p*-values according to the BH procedure, our proposed procedures, and BF procedure. Then we set α = 0.05 and calculate the number rejected null hypothesis (screened features) without adjustment (*p*-value < α) and with adjustment (adjusted *p*-value < α) by each procedure. The mean, and the standard deviation of the number of discoveries for all (r = 1000) replications were calculated separately for each value of ρ.

### Real data application: gene expression data from colon cancer patient tissues

In this section, we evaluate the performance of the proposed procedure as an analysis of a real data set. From the GSE44861 data set of colorectal cancer, we used 111 samples of microarray tests with 22,277 gene expression levels and a binary status feature including 56 samples of cancer tissue (Y = 1) and 55 samples of healthy tissue (Y = 0). This data was generated using the Affymetrix Gene Chip platform and has been preprocessed and the gene expression levels are presented as fragments per kilo base million (FPKM. The normalization process was done using the “edgeR” package in R. This data set is freely available for researchers to investigate gene expression patterns in colon tumors and identify potential biomarkers of colorectal cancer. These data were registered in the GEO database in 2013 and updated in 2017.

Compared to cancerous and non-cancerous cells, if the difference in expression is significant for a specific gene, it can be concluded that the gene was related with colorectal cancer. We used a T-test to find genes associated with colon cancer and to select the significant gene expressions. The hypothesis of this test is as follows,

$$\left\{\begin{array}{l}{H}_{0}:{\mu }_{1i}={\mu }_{2i} \\ {H}_{1}:{\mu }_{1i}={\mu }_{2i}.\quad \forall\, i=1.\cdots .P \end{array}\right.$$

where ${\mu }_{1i}$ is the mean of the ith gene in group 1 (cancerous tissue), ${\mu }_{2i}$ is the mean of the ith gene expression in group 2 (healthy tissue), and P = 22,277. In this way, the *p*-values of the t-tests for all features are determined. Then, we sort the *p*-values in an ascending order. And estimating the bivariate correlation between two consecutive sorted test statistics by calculating the bivariate correlation between their sorted features. Then the adjusted *p*-values based on BF, BH, BY, and three proposed procedures M1, M2, and M3 were calculated and compare with α.

To assessing the efficiency of screen features by different procedures, a multiple logistic regression model was used. Due to quasi complete separation, and small sample size ordinary maximum likelihood approach did not converge. So, the Efficient Bayesian Logistic Regression (EBLR) model that was developed under a highly efficient Ultimate Polya Gamma Marcov Chain Monte Carlo (MCMC) algorithms, was used. The “UPG” package under R4.3.1 was used to fit EBLR model on screened features.

To compare the results of the EBLR model on screened features by different procedures; BF, BY, BH, M1, M2, and M3, we use three approaches:

1. Estimating the Entropy (-log(likelihood)) of models
2. Estimating the Area Under the ROC Curve (AUC) to show the predictive power of models
3. Drawing the box plot for the predicted probability of allocating in the cancerous tissue group (Y = 1) versus the real status (Y = 1/ 0, cancerous or healthy tissue groups) for all models

### Supplementary Information

**Additional file 1: S1.** Descriptive statistics of the number of screened features by BF, BH, BY, M1, M2, and M3 procedures at the different levels of correlation (ρ) in the simulation study for n1=n2=100.

**Additional file 2: S2.** Descriptive statistics of the number of screened features by BF, BH, BY, M1, M2, and M3 procedures at the different levels of correlation (ρ) in the simulation study for n1=n2=30.

**Additional file 3: S3.** 22278 Features (Gene Experissions) of Coloreactal Cancer Data.

**Additional file 4 S4.** R Codes.

## Declarations

### Availability of data and materials

All data were used in this study are from an open access and open sources; the GSE44861 data set of colorectal cancer, this data set was attached at Additional file 3: S3. The R-codes written for this study are available in suppleAdditional file 4 S4.
