# Correction: Modifying the false discovery rate procedure based on the information theory under arbitrary correlation structure and its performance in high-dimensional genomic data

**DOI:** 10.1186/s12859-024-05696-8

**Published:** 2024-02-23

**Authors:** Sedighe Rastaghi, Azadeh Saki, Hamed Tabesh

**Affiliations:** 1 Department of Epidemiology and Biostatistics, School of Health, Mashhad University of Medical Sciences, Mashhad, Iran; 2 Department of Medical Informatics, School of Medicine, Mashhad University of Medical Sciences, Mashhad, Iran

**Correction: BMC Bioinformatics (2024) 25:57** 10.1186/s12859-024-05678-w

Following publication of the original article [1], the authors identified an error in the author name of Sedighe Rastaghi.

The incorrect author name is: Sedighe Rastahi.

The correct author name is: Sedighe Rastaghi.

The author group has been updated above and the original article [1] has been corrected.
