# Supplementary material for: Modifying the false discovery rate procedure based on the information theory under arbitrary correlation structure and its performance in high-dimensional genomic data
Source: BMC Bioinformatics. 2024 Feb 5;25:57. doi: 10.1186/s12859-024-05678-w (PMC10840263; doi:10.1186/s12859-024-05678-w)
Supplement: Supplementary file 1 — Additional file 1: S1. Descriptive statistics of the number of screened features by BF, BH, BY, M1, M2, and M3 procedures at the different levels of correlation (ρ) in the simulation study for n1=n2=100. [file 12859_2024_5678_MOESM1_ESM.docx]

S1: Descriptive statistics of the number of screened features by BF, BH, BY, M1, M2, and M3 procedures at the different levels of correlation (ρ) in the simulation study for n1=n2=100

| ρ |  | Adjustment Procedures | | | | | | |
| --- | --- | --- | --- | --- | --- | --- | --- | --- |
|  |  | Non | BF | BH | BY | M1 | M2 | M3 |
| 0 | Mean | 353.351 | 49.357 | 228.404 | 102.112 | 214.429 | 220.73 | 227.456 |
|  | SD | 11.801570 | 5.802294 | 14.75712 | 11.00773 | 14.25881 | 14.49086 | 15.70872 |
|  | p2.5 | 329 | 39 | 198 | 83 | 201 | 198 | 197.975 |
|  | p25 | 346 | 46 | 219 | 95 | 218 | 219 | 221 |
|  | Median | 354 | 49 | 229 | 102 | 215 | 222 | 229 |
|  | p75 | 361 | 53 | 238 | 109 | 237 | 238 | 240 |
|  | p97.5 | 375 | 60 | 256 | 121 | 255.03 | 256 | 258 |
|  | IQR | 15 | 7 | 19 | 14 | 19 | 19 | 19 |
|  | min | 317 | 34 | 168 | 72 | 166 | 166 | 166 |
|  | max | 386 | 68 | 270 | 135 | 270 | 270 | 273 |
| 0.2 | Mean | 352.212 | 48.633 | 226.385 | 101.588 | 193.784 | 207.094 | 222.139 |
|  | SD | 19.65903 | 9.400994 | 26.03533 | 19.48784 | 24.19663 | 25.12964 | 25.86915 |
|  | p2.5 | 322 | 35 | 187 | 74 | 157 | 169.975 | 183 |
|  | p25 | 339.75 | 43 | 210 | 88 | 178 | 191 | 206 |
|  | Median | 350 | 47 | 222 | 98 | 190 | 203 | 218 |
|  | p75 | 361.25 | 53 | 237.25 | 110 | 206 | 220 | 234 |
|  | p97.5 | 395 | 69.03 | 288 | 146 | 249 | 263.06 | 281.18 |
|  | IQR | 21.5 | 10 | 27.25 | 22 | 28 | 29 | 28 |
|  | min | 303 | 29 | 166 | 64 | 132 | 148 | 161 |
|  | max | 470 | 121 | 396 | 219 | 334 | 364 | 389 |
| 0.4 | Mean | 352.297 | 48.607 | 225.246 | 100.605 | 162.728 | 184.369 | 209.667 |
|  | SD | 33.77996 | 16.18235 | 45.69325 | 33.99483 | 40.78092 | 42.60133 | 44.65537 |
|  | p2.5 | 309 | 30 | 167.975 | 61 | 109.975 | 130.975 | 152.975 |
|  | p25 | 331 | 38.75 | 197 | 79 | 137 | 157 | 182 |
|  | Median | 344 | 45 | 214 | 92 | 154 | 175 | 200 |
|  | p75 | 365 | 54 | 241.25 | 111 | 179 | 200 | 226 |
|  | p97.5 | 438.03 | 88 | 336.09 | 183.06 | 258.03 | 287 | 316 |
|  | IQR | 34 | 15.25 | 44.25 | 32 | 42 | 43 | 44 |
|  | min | 285 | 24 | 139 | 49 | 88 | 108 | 127 |
|  | max | 562 | 174 | 512 | 343 | 415 | 455 | 492 |
| 0.5 | Mean | 351.997 | 48.567 | 225.095 | 100.674 | 147.963 | 171.72 | 201.033 |
|  | SD | 41.92267 | 19.44602 | 56.70119 | 41.75307 | 48.00458 | 51.31983 | 54.32046 |
|  | p2.5 | 301 | 27 | 156 | 54 | 89.975 | 108 | 132 |
|  | p25 | 324 | 36 | 187 | 75 | 116 | 138 | 164 |
|  | Median | 341 | 44 | 210 | 90 | 136 | 160 | 188 |
|  | p75 | 369 | 54 | 248 | 113 | 164 | 190 | 221 |
|  | p97.5 | 456.03 | 97 | 368.03 | 205 | 266.06 | 300 | 340 |
|  | IQR | 45 | 18 | 61 | 38 | 48 | 52 | 57 |
|  | min | 279 | 24 | 141 | 44 | 78 | 90 | 115 |
|  | max | 604 | 198 | 557 | 373 | 460 | 494 | 528 |
| 0.6 | Mean | 352.626 | 48.713 | 223.98 | 100.679 | 133.011 | 157.807 | 188.669 |
|  | SD | 48.74448 | 23.35852 | 65.77831 | 49.88433 | 54.0024 | 58.01539 | 62.06311 |
|  | p2.5 | 294 | 26 | 143 | 48 | 70.975 | 89.975 | 113.975 |
|  | p25 | 320 | 34 | 180 | 69 | 98 | 120 | 147 |
|  | Median | 340 | 41.5 | 206 | 87 | 119 | 143 | 173 |
|  | p75 | 373 | 55 | 250.25 | 114.25 | 152 | 179.25 | 212.25 |
|  | p97.5 | 472.15 | 105 | 384 | 230.03 | 276 | 300.42 | 338.27 |
|  | IQR | 53 | 21 | 70.25 | 45.25 | 54 | 59.25 | 65.25 |
|  | min | 262 | 16 | 118 | 34 | 47 | 65 | 88 |
|  | max | 642 | 241 | 594 | 432 | 479 | 509 | 552 |
| 0.8 | Mean | 351.314 | 48.654 | 222.664 | 100.403 | 101.873 | 124.238 | 153.56 |
|  | SD | 64.60137 | 31.02571 | 86.84043 | 64.95349 | 60.44851 | 67.47738 | 74.74459 |
|  | p2.5 | 277 | 20 | 122 | 38 | 41 | 52 | 70 |
|  | p25 | 309 | 30 | 164.75 | 59 | 63 | 80 | 104 |
|  | Median | 333 | 39 | 199 | 79 | 83.5 | 104 | 133 |
|  | p75 | 376 | 55 | 254 | 119 | 120 | 146 | 180 |
|  | p97.5 | 511.03 | 126 | 439 | 266.03 | 256.03 | 298.03 | 339.06 |
|  | IQR | 67 | 25 | 89.25 | 60 | 57 | 66 | 76 |
|  | min | 250 | 13 | 96 | 25 | 31 | 35 | 48 |
|  | max | 723 | 318 | 687 | 532 | 526 | 561 | 607 |
| 0.9 | Mean | 351.197 | 48.334 | 221.729 | 100.706 | 83.708 | 101.953 | 126.631 |
|  | SD | 73.05033 | 34.77378 | 97.05905 | 73.27475 | 58.18681 | 66.30559 | 74.66513 |
|  | p2.5 | 267 | 18.975 | 109 | 32 | 30 | 37 | 49 |
|  | p25 | 302 | 28 | 155 | 54 | 47 | 60.75 | 78 |
|  | Median | 331 | 37 | 192 | 76 | 66 | 81 | 105 |
|  | p75 | 380 | 54 | 260 | 122.25 | 98 | 123 | 151 |
|  | p97.5 | 530.03 | 137 | 464.09 | 285.09 | 237 | 268.12 | 317 |
|  | IQR | 78 | 26 | 105 | 68.25 | 51 | 62.25 | 73 |
|  | min | 243 | 9 | 84 | 17 | 19 | 25 | 29 |
|  | max | 758 | 353 | 731 | 584 | 505 | 562 | 613 |
| 0.95 | Mean | 350.85 | 48.39 | 220.743 | 100.607 | 72.685 | 86.614 | 106.651 |
|  | SD | 76.59104 | 36.74164 | 102.0592 | 76.72027 | 53.29142 | 60.23118 | 68.18917 |
|  | p2.5 | 261 | 17 | 102 | 29 | 25 | 30 | 38 |
|  | p25 | 298 | 27 | 153 | 53 | 41 | 50 | 64 |
|  | Median | 329 | 36 | 190 | 74.5 | 56 | 68 | 87 |
|  | p75 | 379 | 55 | 261.25 | 122.25 | 83 | 101 | 125 |
|  | p97.5 | 536 | 138.03 | 474 | 301.03 | 208.09 | 242 | 283.06 |
|  | IQR | 81 | 28 | 108.25 | 69.25 | 42 | 51 | 61 |
|  | min | 238 | 10 | 78 | 16 | 14 | 20 | 26 |
|  | max | 768 | 383 | 749 | 596 | 516 | 549 | 582 |
| 0.99 | Mean | 351.06 | 48.43 | 220.587 | 101.008 | 61.414 | 70.063 | 82.937 |
|  | SD | 80.01193 | 38.36541 | 106.2416 | 79.94535 | 43.75739 | 47.44563 | 52.21262 |
|  | p2.5 | 258.975 | 16 | 99.975 | 28 | 23 | 26 | 31 |
|  | p25 | 296 | 26 | 150 | 52 | 35 | 42 | 51 |
|  | Median | 327 | 36 | 189 | 73 | 50 | 57 | 67 |
|  | p75 | 382.25 | 55 | 262 | 123.25 | 69 | 81 | 99 |
|  | p97.5 | 541.06 | 142.15 | 479.12 | 306.06 | 165.24 | 186.03 | 210.06 |
|  | IQR | 86.25 | 29 | 112 | 71.25 | 34 | 39 | 48 |
|  | min | 233 | 8 | 73 | 12 | 13 | 14 | 23 |
|  | max | 782 | 393 | 767 | 611 | 454 | 481 | 504 |

SD= Standard Deviation; p2.5= percentile 2.5; p25= percentile 25; p75= percentile 75; p97.5= percentile 97.5; IQR= Interquartile Range; min= minimum; max=maximum.
