# Supplementary material for: Modifying the false discovery rate procedure based on the information theory under arbitrary correlation structure and its performance in high-dimensional genomic data
Source: BMC Bioinformatics. 2024 Feb 5;25:57. doi: 10.1186/s12859-024-05678-w (PMC10840263; doi:10.1186/s12859-024-05678-w)
Supplement: Supplementary file 2 — Additional file 2: S2. Descriptive statistics of the number of screened features by BF, BH, BY, M1, M2, and M3 procedures at the different levels of correlation (ρ) in the simulation study for n1=n2=30. [file 12859_2024_5678_MOESM2_ESM.docx]

S2: Descriptive statistics of the number of screened features by BF, BH, BY, M1, M2, and M3 procedures at the different levels of correlation (ρ) in the simulation study for n1=n2=30

| ρ |  | Adjustment Procedures | | | | | | |
| --- | --- | --- | --- | --- | --- | --- | --- | --- |
|  |  | Non | BF | BH | BY | M1 | M2 | M3 |
| 0 | Mean | 367.557 | 48.504 | 242.322 | 106.134 | 204.9 | 220.17 | 237.35 |
|  | SD | 11.65644 | 5.37967 | 14.10862 | 10.52515 | 15.61888 | 14.78879 | 15.56179 |
|  | p2.5 | 345 | 38 | 214.975 | 85 | 172.9 | 189.425 | 202.475 |
|  | p25 | 359 | 45 | 233 | 99 | 196 | 211.75 | 229 |
|  | Median | 367 | 48 | 242 | 106 | 204.5 | 220 | 236.5 |
|  | p75 | 376 | 52 | 252 | 113 | 214.25 | 228.25 | 246.25 |
|  | p97.5 | 389.03 | 58 | 268 | 125.03 | 238.12 | 250 | 266.06 |
|  | min | 335 | 30 | 192 | 73 | 161 | 183 | 200 |
|  | max | 410 | 65 | 287 | 145 | 245 | 262 | 280 |
| 0.2 | Mean | 367.405 | 48.496 | 241.548 | 106.107 | 217.185 | 222.568 | 232.805 |
|  | SD | 20.67729 | 9.69484 | 28.55330 | 20.73937 | 26.28634 | 26.81743 | 27.88312 |
|  | p2.5 | 333 | 34 | 193.975 | 74 | 167 | 173.975 | 185 |
|  | p25 | 354 | 42 | 223 | 93 | 201 | 206 | 215 |
|  | Median | 365 | 47 | 239 | 105 | 217 | 221 | 230 |
|  | p75 | 378 | 53 | 257 | 116 | 232 | 238 | 247 |
|  | p97.5 | 409.03 | 70.03 | 300.03 | 152 | 271 | 278 | 291.03 |
|  | min | 311 | 22 | 172 | 56 | 147 | 162 | 169 |
|  | max | 502 | 128 | 434 | 286 | 392 | 407 | 421 |
| 0.4 | Mean | 367.621 | 48.384 | 241.537 | 106.059 | 185.216 | 201.505 | 222.792 |
|  | SD | 35.93938 | 17.04127 | 49.25839 | 37.33585 | 43.30442 | 45.43371 | 47.74313 |
|  | p2.5 | 312 | 25 | 167 | 54.975 | 114 | 129.975 | 147 |
|  | p25 | 344 | 38 | 209 | 82 | 156 | 171 | 191 |
|  | Median | 363 | 45 | 234 | 99 | 182 | 196 | 217 |
|  | p75 | 385 | 55 | 265 | 120 | 208 | 225 | 245 |
|  | p97.5 | 446.03 | 86.03 | 355.06 | 193.03 | 277.03 | 299 | 325 |
|  | min | 279 | 19 | 121 | 33 | 77 | 80 | 95 |
|  | max | 606 | 213 | 568 | 401 | 469 | 506 | 544 |
| 0.5 | Mean | 367.245 | 48.495 | 241.218 | 106.26 | 169.492 | 188.682 | 214.45 |
|  | SD | 44.85491 | 20.65939 | 60.63630 | 46.17589 | 51.09203 | 54.03838 | 57.45726 |
|  | p2.5 | 301.975 | 22.975 | 155 | 48 | 90.975 | 107.975 | 127 |
|  | p25 | 337 | 35 | 201 | 75 | 135 | 153 | 176 |
|  | Median | 361 | 44 | 232 | 97 | 163 | 182 | 205 |
|  | p75 | 387 | 56 | 269 | 122 | 195 | 216 | 243 |
|  | p97.5 | 466 | 97 | 380.03 | 215.03 | 280.03 | 306.09 | 342 |
|  | min | 246 | 14 | 97 | 27 | 47 | 60 | 79 |
|  | max | 683 | 253 | 651 | 474 | 516 | 556 | 593 |
| 0.6 | Mean | 367.552 | 48.43 | 240.474 | 106.528 | 153.983 | 175.676 | 203.467 |
|  | SD | 52.70967 | 24.92939 | 71.39176 | 54.65607 | 57.74725 | 62.26747 | 67.03604 |
|  | p2.5 | 290 | 19 | 135.975 | 39.975 | 70.975 | 84 | 104.975 |
|  | p25 | 332 | 32 | 192 | 69 | 114 | 132.75 | 157 |
|  | Median | 358.5 | 43 | 230 | 97 | 146 | 167.5 | 195 |
|  | p75 | 392 | 57 | 275 | 125 | 182 | 206 | 235.25 |
|  | p97.5 | 490.09 | 110.03 | 410 | 234.09 | 277.12 | 310.06 | 353.03 |
|  | min | 254 | 12 | 86 | 19 | 34 | 39 | 54 |
|  | max | 711 | 291 | 689 | 523 | 551 | 595 | 642 |
| 0.8 | Mean | 367.37 | 48.77 | 239.514 | 107.329 | 123.469 | 144.941 | 173.047 |
|  | SD | 68.87022 | 33.19477 | 94.23788 | 71.89787 | 66.15153 | 73.44847 | 80.70150 |
|  | p2.5 | 268 | 13.975 | 106 | 24 | 35.975 | 46.95 | 62 |
|  | p25 | 320 | 28 | 171 | 59 | 77 | 94 | 116 |
|  | Median | 357 | 40 | 226 | 89 | 111 | 132 | 158 |
|  | p75 | 398 | 58 | 282 | 129 | 152.25 | 177 | 209.25 |
|  | p97.5 | 524.03 | 131 | 463.03 | 286.03 | 276.09 | 315.03 | 360 |
|  | min | 221 | 7 | 54 | 7 | 15 | 17 | 27 |
|  | max | 800 | 375 | 780 | 649 | 596 | 660 | 707 |
| 0.95 | Mean | 367.9 | 48.888 | 238.363 | 107.32 | 94.988 | 110.425 | 131.63 |
|  | SD | 82.1766 | 39.78037 | 111.1797 | 84.62874 | 61.7513 | 68.61907 | 76.31732 |
|  | p2.5 | 249 | 10 | 85 | 13 | 23 | 26.975 | 34 |
|  | p25 | 311 | 24 | 158.75 | 48.75 | 51 | 61 | 77 |
|  | Median | 354 | 37 | 218.5 | 84 | 83 | 98 | 118 |
|  | p75 | 403.25 | 58 | 291 | 135.25 | 121.25 | 141 | 166 |
|  | p97.5 | 555.03 | 145.21 | 497.06 | 320.03 | 239.03 | 271.06 | 309.15 |
|  | min | 203 | 5 | 37 | 3 | 8 | 8 | 9 |
|  | max | 855 | 455 | 849 | 715 | 571 | 618 | 671 |
| 0.99 | Mean | 367.595 | 48.898 | 238.125 | 107.677 | 85.76 | 96.877 | 113.144 |
|  | SD | 85.52434 | 41.70965 | 115.7153 | 88.28138 | 56.45051 | 60.44226 | 65.77449 |
|  | p2.5 | 243 | 9 | 77.975 | 11.975 | 19.975 | 24 | 28.975 |
|  | p25 | 307 | 23 | 155.75 | 48 | 46 | 54 | 65 |
|  | Median | 353 | 37 | 216 | 82.5 | 72.5 | 85 | 103 |
|  | p75 | 406.25 | 57.25 | 291 | 137 | 111 | 126 | 145 |
|  | p97.5 | 561.03 | 151.03 | 506 | 332.03 | 214.03 | 233.06 | 258.03 |
|  | min | 196 | 3 | 30 | 2 | 6 | 6 | 7 |
|  | max | 865 | 471 | 857 | 724 | 521 | 551 | 577 |

SD= Standard Deviation; p2.5= percentile 2.5; p25= percentile 25; p75= percentile 75; p97.5= percentile 97.5; min= minimum; max=maximum.
