# Supplementary material for: Modifying the false discovery rate procedure based on the information theory under arbitrary correlation structure and its performance in high-dimensional genomic data
Source: BMC Bioinformatics. 2024 Feb 5;25:57. doi: 10.1186/s12859-024-05678-w (PMC10840263; doi:10.1186/s12859-024-05678-w)
Supplement: Supplementary file 4 — Additional file 4 S4. R Codes. [file 12859_2024_5678_MOESM4_ESM.docx]

#S4: R Codes

###Required Packages###

library(MASS)

library(pracma)

###Defining the Initial Values###

rm(list=ls())

n1=n2=100

Rho=0.6

p=1000

alfa=0.05

nsim=1000 #number of repetitions

tic("total")

c.p=sum(1/seq(1,p))

###Generating NULL Effect Sizes###

set.seed(2023)

Sigma0=0.0678*diag(p)

delta1=mvrnorm(1,mu=rep(0,p) ,Sigma=Sigma0)

####Generating Covariance Matrix for Composite Symmetry###

Sigma1=1*diag(p) ## covariance matrix

for (i in 1:p){ for (j in 1:p){ if (i<j) {

Sigma1[i,j]=(Rho)

Sigma1[j,i]=Sigma1[i,j] }

} }

####Generating Response Vector###

y1<- matrix(1,n1,1)

y0<- matrix(0,n2,1)

y=rbind(y1,y0)

###Initial Matrix###

corrx1=matrix(0,nsim,p)

pvaluet.test1=matrix(0,nsim,p)

p1=matrix(0,nsim,p)

p.fdr=p.cfdr=p.fdr1=p.fdr2=p.fdr3=matrix(alfa/p,nsim,p)

li1.xd1=li2.xd1=li3.xd1=matrix(1,nsim,p)

gxd11=gxd12=gxd13=matrix(0,nsim,p)

sump.fdr=sump.cfdr=sump.fdr1=sump.fdr2=sump.fdr3=sump.bf=sumpr=0

###SIMULATION###

for(m in 1:nsim){

xd1.sort= matrix(0,n1+n2,p)

xd1=rbind((mvrnorm(n1,mu=delta1 ,Sigma=Sigma1)),(mvrnorm(n2,mu=rep(0,p),Sigma=Sigma1)))

for(i in 1:p){

pvaluet.test1[m,i]=t.test(xd1[,i]~y)$p.value

return

}

###Calculate the adjusted p-value based on FDR and modified FDRs###

isort=data.matrix(c(1:p))

p1[m,]=data.matrix(sort(t(pvaluet.test1[m,])))

xd1.sort=t(data.matrix(sortrows(cbind ((pvaluet.test1[m,]),t(xd1))))[,-1])

for(h in 2:p){

corrx1[m,h]=abs((cor(xd1.sort[,h],xd1.sort[,h-1] , method = "pearson")))

gxd11[m,h]=(1-corrx1[m,h])/(1+corrx1[m,h]);li1.xd1[m,h]=li1.xd1[m,h-1]+(gxd11[m,h])

gxd12[m,h]=1-corrx1[m,h];li2.xd1[m,h]=li2.xd1[m,h-1]+(gxd12[m,h])

gxd13[m,h]=1-(corrx1[m,h]^2);li3.xd1[m,h]=li3.xd1[m,h-1]+(gxd13[m,h])

p.fdr[m,h]=p1[m,h]*p/h

p.cfdr[m,h]=p1[m,h]*p*c.p/h

p.fdr1[m,h]=p1[m,h]*p/li1.xd1[m,h]

p.fdr2[m,h]=p1[m,h]*p/li2.xd1[m,h]

p.fdr3[m,h]=p1[m,h]*p/li3.xd1[m,h]

return}

###Calculate the number of False Discoveries###

sumpr[m]=sum(p1[m,]<0.05)

sump.bf[m]=sum(p1[m,]<0.05/p)

cs=cumsum(p.fdr[m,]<0.05)

csc=cumsum(p.cfdr[m,]<0.05)

cs1=cumsum(p.fdr1[m,]<0.05)

cs2=cumsum(p.fdr2[m,]<0.05)

cs3=cumsum(p.fdr3[m,]<0.05)

sm1=sm2=sm3=sm=smc=p

for(i in 1:(p-1)){

if (cs1[i]==cs1[i+1]) sm1[i]=cs1[i] else sm1[i]=p

if (cs2[i]==cs2[i+1]) sm2[i]=cs2[i] else sm2[i]=p

if (cs3[i]==cs3[i+1]) sm3[i]=cs3[i] else sm3[i]=p

if (cs[i]==cs[i+1]) sm[i]=cs[i] else sm[i]=p

if (csc[i]==csc[i+1]) smc[i]=csc[i] else smc[i]=p

}

sump.fdr[m]=min(sm)

sump.cfdr[m]=min(smc)

sump.fdr1[m]=min(sm1)

sump.fdr2[m]=min(sm2)

sump.fdr3[m]=min(sm3)

}

mean(sumpr);mean(sump.bf);mean(sump.fdr);mean(sump.cfdr); mean(sump.fdr1);mean(sump.fdr2);mean(sump.fdr3)

out=cbind(sumpr,sump.bf,sump.cfdr,sump.fdr,sump.fdr1,sump.fdr2,sump.fdr3)

write.csv(out,"C:/Users/sakia/Desktop/out6.csv")

toc()

proce=c("P","BF","BY","BH","M1","M2","M3")

proc1=as.factor(proce)

plot(t(out)~proc1)
